# Chemical Exploration of Polysaccharides, Fatty Acids, and Antioxidants as Functional Ingredients from Colombian Macroalgae *Acanthophora spicifera*, *Sargassum ramifolium,* and *Sargassum fluitans*

**DOI:** 10.3390/molecules30163333

**Published:** 2025-08-10

**Authors:** Jhonny Colorado-Ríos, Diana C. Restrepo-Espinosa, Yuli Restrepo-Moná, Juan David Monsalve, Diana M. Márquez-Fernández, Leonardo Castellanos, Alejandro Martínez-Martínez

**Affiliations:** 1 Grupo Productos Naturales Marinos, Facultad de Ciencias Farmacéuticas y Alimentarias, Universidad de Antioquia, Calle 70 No. 52-21, Medellín 050010, Colombia; diana.restrepoe@udea.edu.co (D.C.R.-E.); millerlay.restrepo@udea.edu.co (Y.R.-M.); juanmonsalve.mgvc@gmail.com (J.D.M.); diana.marquez@udea.edu.co (D.M.M.-F.); alejandro.martinez@udea.edu.co (A.M.-M.); 2 Unidad de Biología Celular y Molecular, Corporación para Investigaciones Biológicas (CIB), Universidad de Antioquia, Medellín 050010, Colombia; 3 Departamento de Química, Facultad de Ciencias, Sede Bogotá, Universidad Nacional de Colombia, Av. Carrera 30 # 45-03, Bogotá 111321, Colombia; lcastellanosh@unal.edu.co

**Keywords:** macroalgae, Colombia, fatty acids, polysaccharides, polyphenols, antioxidants

## Abstract

Macroalgae are valuable natural sources for bioprospection and the development of raw materials applicable to the nutrition, health, and agriculture industries. To build a basis for the sustainable use of marine organisms from the Colombian Caribbean, a preliminary study was conducted focusing on known functional compounds in two genera of macroalgae, including the species *Acanthophora spicifera* (Rhodophyta), *Sargassum ramifolium,* and *Sargassum fluitans* (Ochrophyta). This study included the extraction and identification of polysaccharides using ultrafiltration, nuclear magnetic resonance (^1^H-NMR), Fourier-transform infrared spectroscopy (FT-IR), and size exclusion chromatography (SEC); fatty acids by gas chromatographic (GC) profiling; and phenolic composition and antioxidant activity by complementary semi-quantitative methods (ABTS, DPPH, FRAP, and ORAC assays). Carrageenan-type polysaccharides were detected in *A. spicifera,* while alginate and fucoidan types were found in *S. ramifolium* and *S. fluitans*; palmitic acid was the predominant fatty acid in *A. spicifera* and *S. ramifolium*, but it was not detected in *S. fluitans*. *S. ramifolium* showed the highest ABTS, DPPH, and ORAC activities and phenolic compounds, while *S. fluitans* exhibited the highest FRAP activity. This study contributes to the chemical knowledge on Colombian macroalgae to establish potential applications in various fields, including biomedicine, cosmetics, functional foods, and nutraceutical ingredients.

## 1. Introduction

Macroalgae are a valuable source of valuable compounds with critical industrial ap-plications, among which polysaccharides (PSs), fatty acids, and antioxidants have commercial recognition with applications in the food, pharmaceutical, and cosmetic industries [1,2] and a promising economic potential for coastal communities where these samples may be collected. The studies about raw material from macroalgae have focused on the use of high value products such as polyunsaturated fatty acids, phenols, and polysaccharides, mainly to be incorporated in foods and as drug delivery systems [3,4].

Alginates and carrageenan, due to their polysaccharide nature, are significant components of seaweed that constitute the cell wall [5]. Alginates have gel-forming properties that are very well appreciated for food, pharmaceutical, and cosmetic applications due to their rheological and suspending properties and their wound-healing effects [6,7,8,9]. Carrageenans are widely used as raw materials for food products such as gelling agents, emulsifiers, thickeners, or stabilizers [10,11]. Brown seaweed commonly present alginates, which are arranged in the linear backbone of blocks composed of β-D-mannuronic acid (M) and α-L-guluronic acid (G) residues, both linked by 1→4 glycosidic bonds [8]. Otherwise, red seaweeds are known for producing polysaccharides mainly composed of galactose, such as carrageenan, that are made by repeating disaccharide units conformed by 3-linked β-D-galactopyranose (G) and 4-linked α-D-galactopyranose (D) or 4-linked 3,6-anhydro-α-D-galactopyranose (DA); these units could be sulphated [12]. The sugar backbone of polysaccharides obtained from different macroalgae families can vary their expected molecular weight, their sulfation substitution and degree, and their biological activities [1].

Carbohydrates are not the only valuable compounds that can be obtained from algae, and although the content of lipids is not as high as in the case of polysaccharides, they represent an alternative source of polyunsaturated fatty acids, especially those belonging to long-chain omega-3 polyunsaturated fatty acids (*n*-3 PUFAs), which traditionally have been obtained from fish or fish oil and are essential in the human diet because of their functionality in cardiovascular and inflammatory diseases, brain development, cancer, anti-aging, and mental health [13,14]. Algae represent a sustainable and non-traditional alternative source of *n*-3 PUFAs since decades of overfishing have significantly declined stocks [14]. While red and brown algae are notable for their eicosapentaenoic acid (EPA) and arachidonic acid (AA) content, green seaweeds primarily contain oleic and palmitic acid [1].

Polyphenolic compounds are also present in macroalgae, such as flavonoids, phenolics acids, phenolic terpenoids, and phlorotannins, the latter being exclusive to brown algae [15,16]. They are essential for the growth and development of these organisms, and they are involved in protective functions since they are produced in response to stimuli that includes predator attacks, diseases, injures, and UV radiation [17,18,19]. Due to these properties, these substances are of interest for developing similar functions that may contribute to healing various diseases, showing a wide range of possible applications in the pharmaceutical and biomedical areas. Among their specific bioactivities, the antioxidant properties of algae are of interest to food industries to improve the nutritional value and sensory characteristics of several processed products such as cheese, yogurt, and desserts [1,20]. Additionally, their cosmetic applications have been investigated by incorporating phenolic compounds from algae, aiming to develop skincare and anti-aging products that leverage their protective effects against the loss of skin elasticity associated with aging [21]. Antioxidant activity is one of the most explored effects of phenolic compounds from macroalgae, but it is not an exclusive property of this type of substance. Other primary and secondary metabolites, such as polysaccharides, chlorophylls, and carotenoids exhibit these properties, which make them extremely valuable bioactive agents that can impact human health through the prevention of oxidative stress and the mitigation of prevalent modern diseases [18,21,22].

Considering the above, it is evident that research on marine algae has become a global trend. However, research remains insufficiently advanced within the Colombian national context to indicate that this area has been thoroughly explored. This may be explained by the fact that, despite having coastlines along both the Atlantic and Pacific Oceans, there is a lack of interest from the national scientific community, multiple coastal regions are challenging and expensive to access, and there are sociopolitical factors that impede access to different regions [23,24]. Additionally, marine macroalgae, as a source of bioactive substances, can be used as a renewable and sustainable natural resource. Nevertheless, several outcomes have demonstrated that the biomass produced by these organisms has a broad spectrum of substances, which varies according to the species and the environmental conditions [1]. Those differences could be evidenced in the explored region when compared with those previously reported in other countries and even continents since Colombia is a transcontinental country situated in the northwest of South America, bordered by the Caribbean Sea and the Pacific Ocean. Simultaneously, investigating these locally occurring organisms may yield novel compounds that have not yet been characterized [23,24].

A bibliographic review conducted through Scopus reveals that, of the 16,223 articles published globally on macroalgae and 32,676 on seaweed, just 23 and 48, respectively, have been produced in Colombia, where around 50% of the research has been focused on agriculture, biological, ecological, and Earth and planetary sciences. At the same time, those related to chemistry, biochemistry, and other fields of knowledge represent a percentage lower than 16%. *Acanthophora spicifera* is an edible red marine alga, widely distributed in subtropical and tropical seas [25], which has been documented as a rich source of carbohydrates, lipids, proteins, minerals, fatty acids, essential amino acids, and functional substances including flavonoids, phenolic compounds, and polysaccharides [26,27]. This species has been mainly studied in the United States and India, countries where environmental conditions differ significantly from those of Colombia, which could suggest that composition may be different among the specimens of this species that are collected in our country.

Regarding the algae of the genus *Sargassum,* growing in the tropical Atlantic Ocean and extending from the Gulf of Mexico to the Gulf of Guinea, they can make massive aggregations in the open ocean and on the coast, which in recent years has conducted negative environmental consequences and has led to the local communities exploring solutions to prevent its accumulation and subsequent decomposition [28,29]. *Sargassum fluitans* is found in the Caribbean’s coastal waters as part of a surface-dwelling species inundating the Caribbean’s shores [30]. Despite its overgrowth, this species acts as a biofilter that could reduce pollution and accumulate toxic metals in the marine environment [31]. In *S. fluitans*, fucoidan extracts have shown antifibrotic and anti-inflammatory effects [32], and organic extracts have shown antioxidant and antibacterial activities [33]. *Sargassum ramifolium* has been less documented, but there are some reports from Caribbean species, more specifically from Cuba and the Yucatán Peninsula [34,35], where they have shown antioxidant activity and the presence of phenolic compounds; however, a complete chemical characterization has not yet been carried out [36].

This research investigates Colombian macroalgae biodiversity, focusing on comparing these algae with those of the same species from different regions. Additionally, a characterization of its promissory substances could represent a strategy to be exploited by the locals since these species can be easily collected. In this way, the methodologies were designed to facilitate extraction in case these results could be transferred to the local communities to contribute to the biomass valorization of Colombian macroalgae as a resource for extracting products with high market potential. The relatively great cost-effectiveness supports this due to the large number of possible target products and the simple processes with reduced operational costs and environmental burdens.

## 2. Results and Discussion

Macroalgae represent an important source of valuable bioactive substances that are promissory for preventing and treating different human conditions. In this way, they are used to develop new food products, nutraceuticals, cosmetics, and other pharmaceutical products of interest. Among those, bioactive substances, minerals, vitamins, essential amino acids, proteins, enzymes, fatty acids, carotenoids, polyphenols, and carbohydrates have been described. These compounds offer excellent benefits that allow humans to use them directly as food or in the production of hydrocolloids, medicines, biofuel, paper, fertilizers, etc. Nowadays, they are considered prized raw materials due to their cultivation, which does not require fresh water, arable land, or fertilizers, and fast growth rates [4].

To build the basis for developing a sustainable use of Colombian biodiversity, a preliminary study of some primary and secondary metabolites in several macroalgae, including *Acanthophora spicifera* (Rhodophyta), *Sargassum ramifolium*, and *Sargassum fluitans* (Ochrophyta), was carried out. Algae samples were collected from El Rodadero (Santa Marta, Colombia), El Cabo de la Vela (La Guajira, Colombia), and Playita Jacobo (Providence, RI, USA), respectively.

### 2.1. Fatty Acids

The fatty acid composition of the seaweed species was explored (Table 1). The total fat content in all species was 0.59, 0.67, and 0.14 per 100 g for *A. spicifera*, *S. ramifolium*, and *S. fluitans*, respectively. The three species showed myristic acid (14:0), palmitoleic acid (16:1ω7), oleic acid (18:1ω9), and linoleic acid (18:2ω6). Other saturated and unsaturated fatty acids identified were linolenic acid (18:3ω3), behenic acid (22:0), and arachidonic acid (20:4ω6). While some polyunsaturated fatty acids (PUFAs) were present, they were not present in high percentage. Also, there was no evidence of important fatty acids such as EPA (eicosapentaenoic acid; 20:5ω3) and DHA (Docosahexaenoic acid; 22:6ω3). Oleic acid was the major fatty acid for *S. fluitans*, the main monounsaturated fatty acid (MUFA) in all species, and was reported as the main MUFA of *A. spicifera* [37]. Meanwhile, linoleic acid was the main polyunsaturated fatty acid (PUFA) in the three species.

As expected, the lipid content in seaweed is low. However, the percentage of different fatty acids was less than the reports; some of them were not even detected. Despite being stored in a controlled environment protected from light and heat, lipid oxidation occurred, which could serve as a basis to explore the rate of degradation during storage and drying processes, thereby helping to evaluate the quality and stability of organic compounds such as fatty acids and even phenolic compounds in the selected algae. Previous reports found lipid oxidation during storage to be low [38]. Although freeze drying yields the highest amount of compounds, oven drying is a better cost-effective alternative than freeze drying [39]. Therefore, freeze drying was not selected to process the samples. Some reports indicate that seaweed species are a balanced source of ω3 and ω6 PUFAs; like those studies, the Colombian analyzed seaweeds contain ω3, ω6 and ω3 and ω9. The algae contain linoleic acid (ω6), which, along with linolenic acid (ω3), is an essential fatty acid that must be obtained through diet, but linolenic acid was only present in *S. ramifolium*. In previous research, fatty acids like EPA (ω3), arachidonic acid (ω6), oleic acid, and palmitic acid have shown the highest percentage in total FAME analysis; in this case, eicosapentaenoic acid was not detected, and palmitic acid was the main component of the analyzed FAMEs, but it was not detected in *Sargassum* spp. Arachidonic acid was not detected in *A. spicifera*. Oleic acid is present in the three species, mainly in *A. spicifera* and *S. ramifolium* [37,40].

The fact that the studied algae contain some of the reported fatty acids could validate their use as a nutritional source since PUFAs and MUFAs are needed for normal growth and development, neuronal activity regulation, cellular function and signaling, obesity prevention, immune response, and hypertension regulation, among others [40,41,42]. However, it is mandatory to go beyond these results to explore other nutritional components like proteins, vitamins, amino acids, and minerals; these are the reason why they have been consumed as food in Asian countries since ancient times and might be presented to regional communities as a food source, as they are produced in large quantities during various seasons [43]. This study aimed to detect relevant concentrations of distinctive fatty acids commonly found in macroalgae, including the presence of specific long-chain polyunsaturated fatty acids (PUFAs), such as EPA, DHA, and arachidonic acid (ARA), which have recognized nutritional, biomedical, and industrial value. Even at low concentrations, the identification of fatty acids in macroalgae contributes valuable insights into their biochemical potential, ecological role, and commercial relevance.

### 2.2. Polysaccharides

From about 5 g of dry mass of each macroalgae, 17, 6.3, and 4.9% of total polysaccharides were obtained from *A. spicifera*, *S. ramifolium,* and *S. fluitans*, respectively. The dried extracts were subjected to FT-IR analysis. The spectra of the aqueous extract obtained from the three species present typical carbohydrate bands related to hydroxyl (3600–3050 cm^−1^), methylidyne or methine (3050–2800 cm^−1^), carbonyl groups (1700–1400 cm^−1^), C-O/C-C (1200–800 cm^−1^) bonds, and pyranoses or their derivatives (fingerprint region) [44].

The specific bands are different when comparing the spectrum of the extract of *A. spicifera* with those obtained from *Sargassum* spp.; the main difference is associated with the prominent band between 1220 and 1260 cm^−1^, which can be related to sulfation [44,45,46,47].

**Figure 1:**
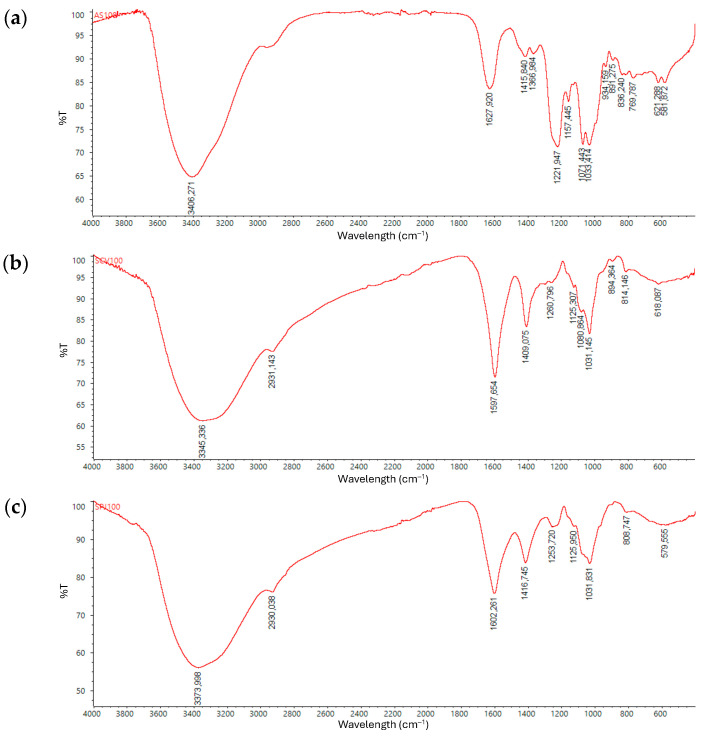
FT-IR spectra of polysaccharides in aqueous extracts from (**a**) *A. spicifera*, (**b**) *S. ramifolium,* and (**c**) *S. fluitans*.

In the infrared spectrum of the aqueous extract from *A. spicifera* (Figure 1a), a broad band at 3406 cm^−1^ was assigned to hydroxyl group O-H stretching vibrations. A similar band is also present in the spectra obtained from both *Sargassum* algae (Figure 1b,c) as well as a weak band around 2930 cm^−1^ that corresponds to the C-H stretching vibrations and two bands related to the asymmetric and symmetric stretching vibrations of carboxylate group O-C-O around 1600 and 1400 cm^−1^ [48,49,50].

Polysaccharides in the aqueous extract from *A. spicifera* showed signals commonly present for carbohydrates such as 1221, 1157, 1071, 934, and 891 cm^−1^ bands. The band at 1072 cm^−1^ is attributed to the main structure of galactan-type polysaccharides reported previously for red algae, which are producers of galactans like agarans and carrageenan (Figure 1a) [26,51,52]. Additionally, a band related to 3,6 sugar is observed at 931 cm^−1^, and this is also consistent with the fact that 3,6-anhydrogalactose (3,6-AG) is a component of polysaccharides such as galactans [52,53].

Additionally, the bands found at 1249 and 891 cm^−1^ are related to a sulfated agar due to corresponding to the sulfated and agar groups, respectively. This is supported by the band at 836 cm^−1^ assigned to 2-O-sulfate of the D-galactose residues [26,44,47]. A similar band around 1260 cm^−1^ is associated with the asymmetric S=O stretching vibrations in the sulfuric acid group that appears in the spectra obtained from *Sargassum* spp. The band around 840 cm^−1^ is only present in the *S. fluitans* spectrum. These differences might suggest that the aqueous extraction from *A. spicifera* allowed it to obtain a polysaccharide with prevalent sulfation. A low intensity band at 1157.445 cm^−1^ in this spectrum can correspond to the C-OH deformation of secondary alcohol or to -SO_3_ asymmetric stretching, as it has been previously reported for sulfated carbohydrates. The high-intensity band at 1033.414 cm^−1^ can be related to C-O deformation and vibration of the pyranose rings; this band is also present in *Sargassum* spp. extracts [48,54]. At 1366 cm^−1^, a low-intensity band can also correspond to C-C-H and O-C-H deformations, which is not present in the spectrum of the other extracts [50].

At the fingerprint or so called anomeric region between 950 and 750 cm^−1^, common bands discussed in carbohydrates such as alginate, fucoidan, carrageenan, and agaran are observed. In this way, bands around 814 and 808 cm^−1^ in polysaccharides obtained from *Sargassum* spp. have been associated with the presence of hexuronic acids such as mannuronic or guluronic acid residues, which can suggest that polysaccharides obtained from *Sargassum* spp. contain acid sugars, as reported in different characterization studies for this genus [44,48,50,54,55]. A band near 1125 cm^−1^ is present in the IR spectrum of the polysaccharides obtained from *S. fluitans* and *S. ramifolium* and it is also related to C-O stretching vibrations [48,54].

Based on the signals reported in various analyses of algal-derived polysaccharides, it is possible to identify the structural features of mixtures of polysaccharides using a single infrared spectrum, even exhibiting overlapping signals that are not necessarily associated with the same structural features. The bands 3374, 2930, 1602, 1417, 1254, 1032, and 809 cm^−1^ in the IR spectrum of the *S. fluitans* polysaccharide is consistent with the previously reported signals in a fucoidan extract from *S. fluitans* Borgesen, collected off the coast of Puerto Morelos, Mexico [32]. This analysis suggests that polysaccharides from these raw materials can be extracted through simple aqueous extraction with ultrafiltration steps.

**Figure 2:**
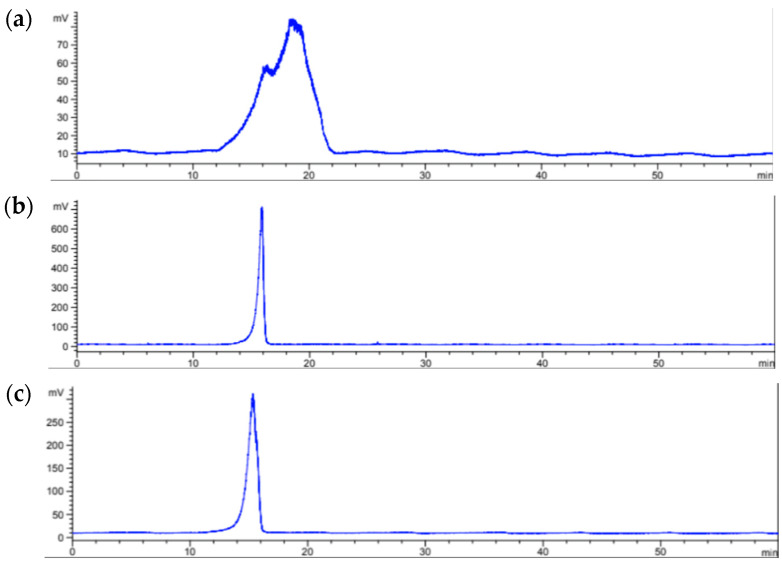
Size exclusion chromatograms of (**a**) *A. spicifera*, (**b**) *S. ramifolium,* and (**c**) *S. fluitans*.

Through chromatographic separation (Figure 2) by size exclusion chromatography, it was possible to observe that the extracts of *Sargassum* species showed a sharp peak while the extract of *A. spicifera* showed two broad peaks, suggesting the presence of heterodisperse polysaccharides, which could explain the number of signals in the infrared spectrum of this algae species.

The average molecular weight of the polysaccharides obtained from the three species of algae was estimated using a standard curve with pullulans of different nominal molecular weight (MW, kDa), including P10 (9600), P50 (47,100), P100 (107,000), and P200 (194,000). Thus, an equation was used to assign an approximated molecular weight to the polysaccharides present in the macroalgae according to their retention time (Table 2).

The polysaccharides present in the aqueous extract of *A. spicifera* have a molecular weight between 92 and 160 kDa. *S. ramifolium* apparently possesses a single polysaccharide with a uniform distribution of weight near 176 kDa, similar to one of those found in *A. spicifera,* while the polysaccharide from *S. fluitans* presents the highest weight (185 kDa), larger than the molecular weight of the previously reported fucoidan of 60 kDa [32]. As it is known, the high molecular weight of polysaccharides from algae make them interesting to incorporate into different formulations, such as cosmetics and alimentary, mainly due to their capacity to be used as carriers or gelling agents, as well as actives with several pharmacological effects (antioxidant, anti-inflammatory, immunomodulator, antitumor) [56]. Other potential advantages of these carbohydrates is their biocompatibility and bioavailability, which allow them to be used in different matrices and applications [57,58,59,60].

Polysaccharides that are typically synthesized by seaweed possess a distinct chemical composition that may differ according to several factors, such as the species’ life cycle, environmental conditions, and even the extraction techniques [61]. Comprehensive structural characterization of algal polysaccharides requires highly purified samples to allow for the accurate determination of their monosaccharide composition, substitution patterns, and sulfate content, among other features. Since direct interpretation by ^1^H-NMR was not enough to encompass those multiple features, it was decided to compare the proton spectrum of the carbohydrates present in aqueous extracts with the obtained spectra from standard algae polysaccharides such as fucoidan, alginate, and carrageenan as well as with other polysaccharides previously reported in several studies. The ^1^H-NMR spectra obtained for the extracts of the three algae show signals with chemical shifts in a spectral range from 6.0 to 3.0 ppm, which is expected for carbohydrates. In addition, some of them present typical signals from the chemical groups attached to the sugar rings around 1.0–2.0 ppm, which are commonly correlated with methyl protons located in different positions of the backbone.

**Figure 3:**
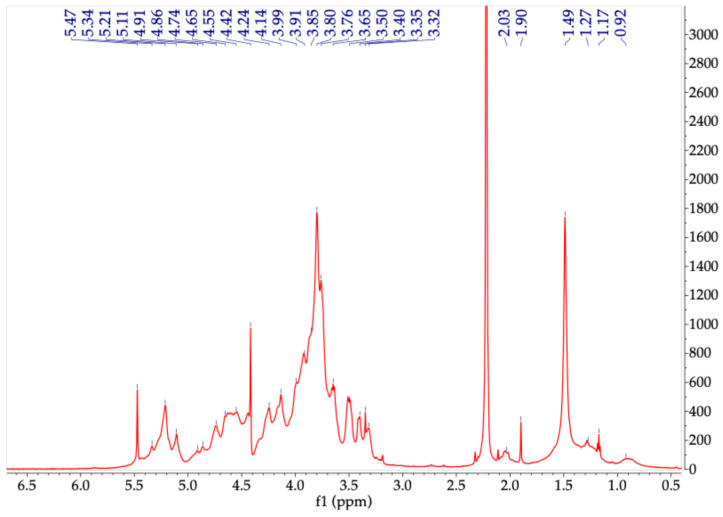
^1^H-NMR spectrum of aqueous extract of *A. spicifera;* recorded at 60 °C with acetone (2.22 ppm) as internal standard. Sample dissolved in deuterium oxide.

Although the acquired spectra of the carbohydrate extract obtained from the red algae *A. spicifera (*Figure 3)*,* did not enable homo- and heteronuclear correlation analyses, many observed chemical shifts were consistent with previously reported values for monomeric units found in various types of carrageenans and agarans, as noted in red seaweed studies [61]. It is possible to observe chemical shifts in the anomeric signals between 5.10 and 5.47 ppm like those that appear in the spectrum obtained for carrageenan, which can correspond to α-D-galactopyranose and its 3,6-anhydro-derivative. Other anomeric signals appear at around 4.4 and 5.0 ppm; however, they show expected overlapping. There are ^1^H-NMR chemical shifts (ppm) consistent with those reported for the DA (4-linked 3,6-anhydro-α-D-galactopyranosyl) unit typical for carrageenans which includes H-1 5.11, H-2 4.15, H-3 4.55, and H-5 4.65 ppm. Furthermore, they are coincident with chemical shifts (H-1 5.49, H-2 3.60, H-3 3.95, H-4 4.22, H-5 4.18, H-6 3.95–3.82) related to L2M (4-linked 2-O-methyl-α-L-galactopyranosyl) units that can be present in agarans. The anomeric proton at 5.34 ppm and other observed signals have been reported for L (4-linked α-L-galactopyranosyl) monomers, the usual constituents of agarans [62,63]. Different from the carrageenan standard, an intense signal at 1.49 ppm suggests the presence of methyl groups along the main backbone [62]. This signal has been related to the 4,6-O- (1′-O-carboxyethylidene) pyruvate group present in an agar-type polysaccharide of high molecular weight previously obtained from *A. spicifera*, suggesting that the main polysaccharide of this extract possesses a characteristic structure of agaran. In addition, this polymer can be composed using small amounts of other monomers, such mannose, arabinose, or xylose, as substituents of its main galactose backbone [26,64,65].

**Figure 4:**
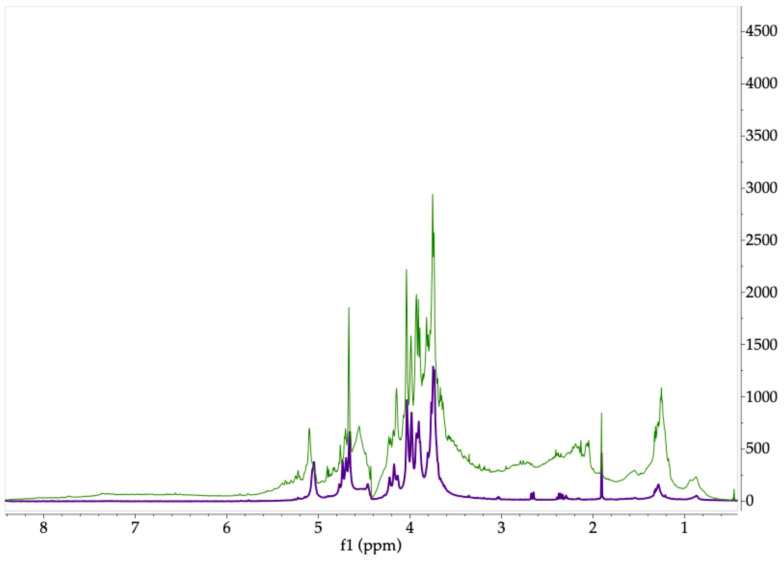
Comparison of ^1^H-NMR spectra of commercial alginate (purple) and aqueous extract of *S. ramifolium* (green). Samples dissolved in deuterium oxide.

*Sargassum* spp. are rich in diverse polysaccharides with physicochemical characteristics that identify them as phycocolloids, which include fucoidan, alginate, and laminarin. They are recognized for exhibiting neuroprotective, antitumor, antimicrobial, and antioxidant effects [66,67]. When the signals in the spectrum of the aqueous extract obtained from *S. ramifolium* were compared with those of the alginate standard (Figure 4), a proton spectrum with similar chemical shifts was observed, alluding to the presence of an alginate-type polysaccharide in the analyzed extract. Thus, the observed spectrum displayed signals corresponding to the anomeric protons of guluronic (G) and mannuronic (M) acid units, which have a chemical shift of around 5.1 ppm and 4.67 ppm, respectively. At the region between 4.67 and 4.76 ppm, the frequencies of the diads and triads of uronic acid blocks can be observed; for example, diads such as MM and MG and triads like MGM have been reported around 4.65, 4.68, and 4.70 ppm [68,69]. The region between 4.4 and 3.6 ppm displays multiplets corresponding to the H-2, H-3, and H-4 protons of the ring of both mannuronic and guluronic acid residues. Additionally, low intensity signals in this region can be attributed to minor amounts of hexoses, indicating the possible presence of other neutral sugars but in lower proportions [50,55,69].

**Figure 5:**
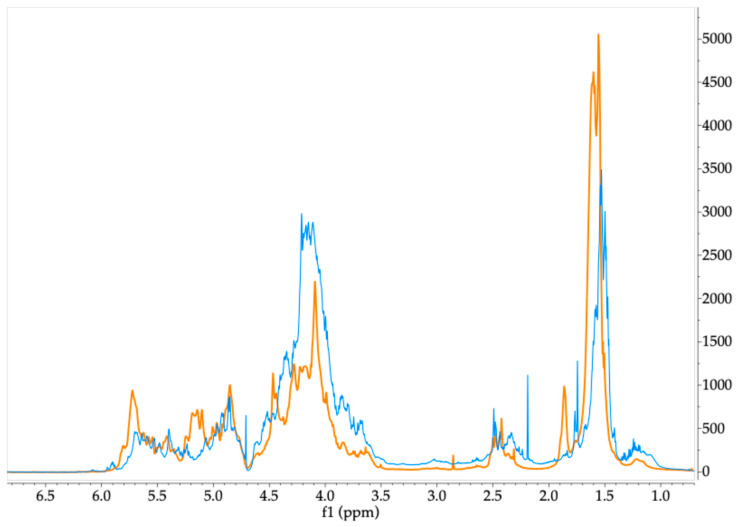
Comparison of ^1^H-NMR spectra of commercial fucoidan (orange) and aqueous extract of *S. fluitans* (blue). Samples dissolved in deuterium oxide.

On the other hand, the ^1^H-NMR spectrum of the algal extract obtained from *S. fluitans*, shown in Figure 5, displays the characteristic proton signals of fucose in the 1.0–1.6 ppm range, corresponding to the methyl groups (H-6) of this monosaccharide. The strong intensity of these signals suggests that fucose is a major component of the polymer backbone, supporting observations from comparisons between the algal spectrum and that of a fucoidan standard. This polysaccharide is claimed as the primary bioactive compound found in *Sargassum* species [66]. Additionally, the chemical shift in the signals in this range varies, pointing to alternating linkages or a different substitution pattern of fucose residues, including sulfate groups. The presence of signals within the ranges of 4.5–5.7 ppm has been attributed to the anomeric protons of fucoidan, where 4.35 ppm is reported to sulfate fucopyranose, and signals around 5.1 and 5.7 ppm could be assigned to α-L-fucopyranoside rings [55,70]. Finally, a chemical shift around 2.2 ppm can be observed as a signal associated with the methyl protons of O-acetyl groups, and signals of the ring region between 3.6 and 3.85 ppm can be related to other monomers like hexoses (galactose, mannose, glucose) and xylose [70,71].

Following this carbohydrate analysis, it was possible to demonstrate through various techniques that the aqueous extracts from the different algae exhibited distinct compositions. This is consistent with the observations made in the acquired spectra, based on previously reported signals in the literature or through comparison with different standards.

### 2.3. Polyphenols and Antioxidant Activity

Different macroalgae are sessile organisms subjected to high UV radiation and temperatures, conditions that make them develop defense mechanisms such as antioxidants, which allow them to prevent, scavenge, and repair systems. This antioxidant capacity is an important tool against oxidative stress as an imbalance of free radicals and antioxidants in the body leads to cell damage related to many diseases [72]. Extensive exposure to stress, the sun, pollution, and toxins can cause the body to increase the level of free radicals, and several natural products rich in antioxidants could help to counteract their effect. Macroalgae have been shown to contain potent natural antioxidant compounds known as polyphenols, which act against oxidative damage and possess biological effects such as being anti-diabetic, anti-allergic, anti-inflammatory, anti-microbial, anti-viral, anti-photoaging, and anticancer [64,73].

In order to evaluate the phenolic content and its antioxidant effect, the extracts of the macroalgae were tested using Folin–Ciocalteu reagent to estimate the total phenolic compounds (TPCs) and by different assays, known as the 3-ethylbenzothiazoline-6-sulfonic acid (ABTS) assay, ferric reducing ability of plasma (FRAP) assay, 2,2-diphenyl1picrylhydrazyl (DPPH) radical scavenging capacity assay, and oxygen radical absorbance capacity (ORAC) assay, for the antioxidant effect [74]. The results of polyphenol and antioxidant activity are summarized in Table 3.

#### Total Phenolic Compounds and Antioxidant Potential

The red seaweed *A. spicifera* presents the lowest total phenolic content (366.08 mg GAE/100 g dw), followed by the brown seaweed *S. ramifolium* (450.30 mg GAE/100 g dw). The highest TPC was observed in the brown alga *S. fluitans* (936.79 mg GAE/100 g dw) (Table 3). Although the Folin Ciocalteau assay is commonly used to determine phenolic compounds content, it is known that other oxidizable groups interact with the reagent, so these results also suggest that some of the compounds present in the algae have a reduction capacity, which directly correlates with antioxidant activity [75]. Substances such as pigments (carotenoids), chlorophylls and derivates, and polysaccharides have been shown to interact with the Folin–Ciocalteu reagent, contributing to the positive result of this test. They also enhance radical scavenging potential when mixed with other antioxidant compounds [76,77]. Since TPC analysis was conducted using aqueous extracts, the presence of water-soluble compounds may have led to an overestimation of the phenolic content, and the actual concentration of phenolic compounds in each algal species could be lower. Previous studies have reported the TPC values for the same species. For instance, *S. ramifolium* has shown TPC values ranging from 6.86 to 29.2 mg GAE/g dw [78], while *A. spicifera* exhibited values between 0.109 and 0.941 mg GAE/g dw [79]. In the case of *S. fluitans*, the presence of phenolic compounds and flavonoids has been confirmed; however, direct comparison with the current study is not possible due to differences in the sample preparation. Previous reports indicated TPC values of 62 mg GAE/g in acetone extract and 69 mg quercetin equivalents/g in ethanol extract [33]. The process should be validated to establish the reported impact of the storage time in declining the content of phenolic compounds and antioxidant activities in some *Sargassum* species [80].

The reducing capacity was evaluated by the FRAP method, which was also based on a SET reaction and possessed high sensitivity and precision [75]. A comparison between the TPC results and the subsequent data obtained by the FRAP assay revealed an inconsistent trend, wherein the sample with the highest reducing capacity did not exhibit a direct correlation with the concentration of phenolic compounds. Nevertheless, the red alga *A. spicifera* presented the lowest values in both tests.

Considering that the ABTS assay is not highly specific for phenolic compounds and may be influenced by the presence of other antioxidants, such as carotenoids, as well as vitamins E and C, it is advisable to verify the results using the FRAP method, which offers greater specificity for phenolics [75]. However, the ABTS values align with the TPC assay outcomes, where *S. ramifolium* has the highest scavenging activity toward this free radical. Another free radical to test scavenging capacity is DPPH; this sample also yielded results consistent with those of the TPC analysis, as evidenced in the previous assay.

A comparative analysis of the results obtained from the ABTS and FRAP assays provides insights into the approximate carotenoid content present in the studied extracts. The necessity of employing both methods becomes particularly evident in the case of the *S. fluitans* and *S. ramifolium* species. While the ABTS assay indicates a high antioxidant capacity in these samples, the FRAP assay reveals significantly lower antioxidant levels. This result suggests substantial amounts of non-phenolic antioxidant compounds, such as carotenoids, which are more effectively detected by the ABTS method. Consequently, these samples are likely rich in other antioxidant compounds that are not efficiently quantified by either method alone.

Through the oxygen radical absorbance capacity (ORAC) determination, the species *S. ramifolium* once again demonstrated the highest activity with a media of 3363.65 mg Trolox/100 g dw, indicating a strong peroxyl radical scavenging capacity. This high antioxidant activity is likely attributable to its levels of phenolic compounds, as previously observed in the TPC assay [81]. This ORAC value was followed by *S. fluitans* and *A. spicifera*, which also demonstrated considerable antioxidant activities, though to a lesser extent (1898.79 and 541.39 mg Trolox/100 g dw, respectively). Additionally, the outcomes observed in the ORAC assay closely matched the trends identified in the ABTS and DPPH assays, reinforcing the consistency of antioxidant profiles across different methods.

After conducting normality and homogeneity of variances tests, the findings from each of the assays were statistically analyzed using a one-way ANOVA test; the results are presented in Table 4.

Given that the one-way ANOVA indicated statistically significant differences across all assays, a post hoc analysis was subsequently conducted using Tukey’s Honest Significant Difference (HSD) test to identify pairwise differences between groups (Table 5).

Post hoc analysis using Tukey’s test revealed statistically significant differences between all the groups since comparisons yielded *p*-values below 0.05, confirming that the observed differences were unlikely to have occurred by chance. The most pronounced difference was observed between *A. spicifera* and *S. ramifolium*, with a mean difference of 2822.26 units, indicating a substantial increase in the measured ORAC assay in *S. ramifolium*. Additionally, a significant but more moderate difference was found in the FRAP test between *A. spicifera* and *S. fluitans*, with *A. spicifera* exhibiting a mean value that was 15.41 units lower than that of the other algae.

The seaweed *A. spicifera* has been described as a natural source of antibacterial, antitumoral, antiproliferative, procoagulant, and antioxidant substances such as phenolic compounds, flavonoids, and polysaccharides [26,64]. For instance, a flavonoid rich fraction were tested on rats, and an anti-diabetic action were observed, which is related to the anti-oxidative action of the flavonoids [82]. This algae also has bromophenols, tannins, and terpenoids that exhibit biological activities such as anticancer, anti-inflammatory, antiproliferative, antimicrobial, and antioxidant activities [83,84]. Additionally, it has been reported that sulphated polysaccharides from this species possess a reducing effect and scavenging activity against DPPH radicals, which strengthens the notion that carbohydrates, along with phenols, may contribute to enhancing the antioxidant effect [64,85]. Similarly, polysaccharides extracted from *Sargassum* macroalgae have also shown antioxidant effects. Fucoidan, for example, has shown activity both alone and in synergy with others against free radicals such as DPPH or in reducing the ferric ion in the FRAP assay. On the other hand, alginate is an antioxidant agent that has already been used as an active agent and as a carrier in different formulations [66]. Although there are not many reports related to the antioxidant effect of the studied *Sargassum* species, carotenoids such as β-carotene and canthaxanthin obtained from other species have been shown to be potential antioxidants [86]. At the same time, seaweed extracts containing sodium alginate and carrageenan increased the activity of some enzymes or cells that were the key indicators of antioxidant status [76]. However, it has been demonstrated that the antioxidant activity of fucoidan extracted from *Fucus vesiculosus* was exerted by the co-extracted phenolic compounds (e.g., phlorotannins) or terpenoids (e.g., fucoxanthins), which are truly responsible for the antioxidant effects rather than fucoidan itself [87]. Co-extraction of phenolic compounds with fatty acids and terpenoids is expected when using methanol to prepare the extracts for the DPPH, FRAP, and ABTS tests and, to a lesser extent, with oligo or polysaccharides (fucoidan or carrageenan) when water is employed as a solvent for TPC and ORAC tests. Accordingly, antioxidant effects involve compounds with different reactivities and solubilities extracted specifically from each sample being screened, requiring a more specific, structural, multifactorial, and quantitative approach.

All findings align with previously reported studies and reinforce the understanding that marine macroalgae are a valuable natural source of antioxidant agents, consistent with the existing scientific literature. Both brown and red algae have been shown to contain a variety of bioactive compounds with notable antioxidant properties. Specifically, the substantial mean difference observed between certain groups supports the presence of compounds different from polyphenols such as pigments, tocopherols, and other non-phenolics that display a marked antioxidant effect [28,51,88,89].

## 3. Materials and Methods

### 3.1. Sample Collection

The algae samples were collected in different regions of Colombia, including the La Guajira, Providencia, and Magdalena regions (Table 6). The *Sargassum ramifolium* sample was obtained from El Cabo de la Vela (12°12′22.20″ N, 72°9′21.96″ W), *Sargassum fluitans* from Playita Jacobo (12°35′36.69″ N, 81°42′28.44″ W), and *Acanthophora spicifera* from El Rodadero (11°7′46.73″ N, 74°13′48.79″ W). All the specimens were collected and supported with environmental permission granted by the Ministerio de Ambiente y Desarrollo Sostenible through contract 121 of 22 January 2016 (otro-sí Nº 7), which regulates research permission of marine samples. The samples were collected by scuba diving conducted by biologists Monica Puyana Hegedus, Brigitte Gavio, and Felipe De La Roche Zogby. A sample of each species was identified and registered for preservation and comparison under the code in Table 6, in the collection of Instituto de Ciencias Naturales, Universidad Nacional de Colombia (Herbario JIWUKORI).

From each sample, foreign material was removed by washing with seawater. Then, the macroalgae were left exposed to the sun and dried in an oven at 40 °C for 48 h. The dried algae were ground, and the resulting material was stored protected from light and heat.

### 3.2. Reagents

Alginic acid ref. A3249 was purchased from PanReac AppliChem (Saint Louis, MO, USA); carrageenan and fucoidan were obtained from Sigma (Saint Louis, MO, USA). With the exception of the ethanol used to wash the algae (96%, special purity, L&F, Medellin, Colombia), the solvents were of analytical grade (Merck, Darmstadt, Germany). The reagents used in the different assays were Folin–Ciocalteu reagent (analytical grade; Merck), sodium carbonate (>99%; Merck), potassium persulfate (>99%; Merck), 2,2′-azino-bis(3-ethylbenzothiazoline-6-sulfonic acid) diammonium salt (ABTS; >98%; Merck), and 2,2-diphenyl-1-picrylhydrazil (DPPH; >99%; Merck). For the antioxidant tests, gallic acid (>99%; Merck) and 6-hydroxy-2,5,7,8-tetramethylchroman-2-carboxylic acid (“Trolox”; >97%; Merck) were used as reference standards.

### 3.3. Fatty Acid Analysis

The dried and ground algae were analyzed by preparing methyl esters of fatty acids using BF_3_ in methanol according to the AOAC Official Method 996.06 -Fat (Total, Saturated, and Unsaturated) in Foods: Hydrolytic Extraction Gas Chromatographic Method [90,91]. In total, 0.5 g of dry macroalgae sample was mixed with 2 mL of ethanol 96%, 100 mg of pyrogallic acid, and 2.0 mL of glyceril-triundecanoate (4.982 mg/mL) as internal standard, followed by the addition of 10 mL of hydrochloric acid (8.3 M) and heating at 70 °C for 40 min, in a tightly capped tub. The fatty acids were extracted with 10 mL ethanol, 25 mL ethyl ether, and methylated using 7% BF_3_ in methanol at 100 °C (in a dry block) for 45 min. The phase containing fatty acid methyl esters (FAMEs) was dehydrated with Na_2_SO_4_ and anhydrous sodium sulfate and transferred to 1.5 mL vials for capillary gas chromatography coupled with mass spectrometry (GC-MS) in a Shimadzu (GCMS-TQ8050 NX) equipped with a ZB-FAME capillary column (30 m length × 0.25 mm i.d. × 0.2 μm). The oven temperature was held at 100 °C for 2 min, increased to 250 °C at a rate of 3 °C/min, and then held at 260 °C for 2 min. The injector and ion source temperatures were 250 °C and 260 °C, respectively. One microliter of each sample was injected with a split ratio of 50:1. The carrier gas was nitrogen at a constant flow rate of 0.71 mL/min. The areas and retention times of FAMEs in the samples and in the standard solution of mix FAMEs (Supelco 37 Component FAME Mix) were quantitatively measured against the C_11:0_ internal standard (incorporated into each sample and standard vial) (See Supplementary Materials). Values are given as grams of fatty acid in 100 g of dry weight sample. Fatty acids below 0.01 g/100 g DW were regarded as not detected.

### 3.4. Carbohydrate Analysis

#### 3.4.1. Aqueous Extract

Before polysaccharide extraction, the dried seaweeds were incubated overnight in an 80% ethanol solution at room temperature. The pretreated biomass was then filtered and rinsed with distilled water. Polysaccharides were subsequently extracted from 5 g of seaweed through four successive extraction cycles, each involving 100 mL of distilled water. Seaweeds of the *Sargassum* genus were submitted to heating (60 °C) and stirring (100 rpm) while the extract of *A. spicifera* was obtained by an ultrasound-assisted extraction using a PEX3 Sonifier (REUS, Contes, France) at 60 °C and 80 Hz. Finally, the aqueous extracts were diluted with water until a fluid suspension and ultrafiltered using a Sartorius Vivaflow 200 system with a 10 kDa molecular weight cutoff membrane followed by a 100 kDa membrane. Retained extracts in the 100 kDa membranes were freeze dried and then used for structural analysis.

#### 3.4.2. FT-IR Analysis

Infrared spectroscopy was used to identify the presence of carbohydrates in the aqueous extract obtained from *S. ramifolium*, *S. fluitans,* and *A. spicifera*. FT-IR spectra of lyophilized extracts were acquired with a Nicolet™ Summit™ 912A1139, with an attenuated total reflectance (ATR) accessory; measurements were performed at 20 °C in a range from 4000 to 400 cm^−1^, with a resolution of 4 cm^−1^ and 16 scans.

#### 3.4.3. Size Exclusion Chromatographic Analysis

Chromatography was performed using an Agilent 1100 HPLC (Waldbronn, Germany) system equipped with a UV detector set at 280 nm, followed by an evaporative light scattering detector (ELSD; Varian 380-LC, Shropshire, UK). A Superose 12 10/300 GL (GE Healthcare, Uppsala, Sweden) column (11 µm, 300 mm × 10 mm) was used for separation, and elution was carried out with water at a flow rate of 0.5 mL/min for 60 min. A pullulan standard kit (Standard P-82, Shodex, Kawasaki, Japan) with various molecular weights was used to estimate the average molecular weight of the polysaccharides in the aqueous extracts.

#### 3.4.4. ^1^H-NMR Analysis

The proton NMR spectra of the extracts were recorded on a 600 MHz Bruker Avance III (Ettlingen, Germany) by dissolving 10 mg of extract with 0.75 mL of D_2_O (99.9%) and transferring it to an NMR tube. Spectra were acquired at 60 °C, a spectral width of 3000 Hz, 64 scans, and a 90° pulse; acquisition mode = 2 s, recovery = 5 s (for a complete return after a 90° pulse). Acetone was used as internal reference for the extract from *A. spicifera* because the standard was not available for comparing chemical shifts.

### 3.5. Total Phenolic Compounds and Antioxidant Potential Assays

#### 3.5.1. Total Phenolic Compounds (TPC)

TPCs were determined using the Folin–Ciocalteau method of the AOAC 2017.13 [92]. The sample of each alga was prepared by mixing 1.0 g of algae with 100.0 mL of water. An aliquot of 50 μL from the sample solution was mixed with 100 μL of Folin–Ciocalteau reagent, 300 μL of 20% sodium carbonate solution, and 15 mL of deionized water. The resulting mixture was incubated in the darkness at 25 °C for two hours, and its absorbance at 765 nm was measured in a spectrofluorophotometer (Synergy H1, Biotek^®^, Winooski, VT, USA). Methanol was used as a negative control. The total phenolic content in each algae extract was determined using a standard curve of gallic acid (40–200 mg/L), and results were expressed as milligrams of gallic acid equivalents (GAE) per 100 g of dried sample.

#### 3.5.2. Antioxidant Assays

##### ABTS Assay

The ABTS assay was conducted by mixing 10 μL of a sample solution (500 mg of dry sample in 10 mL of methanol) with 190 μL of a 3.5 mM solution of ABTS. The absorbance of this mixture was acquired at 732 nm after being incubated at room temperature in darkness for 30 min. The ABTS radical-scavenging activity was estimated through the Trolox Equivalent Antioxidant Capacity (TEAC) value [93].

##### DPPH Assay

The DPPH test was performed by mixing 10 μL of a sample solution (500 mg of dry sample in 10 mL of methanol) with 190 μL of a 200 ppm solution of the 2,2-diphenyl-1-picrylhydrazyl (DPPH) radical. This mixture was incubated at room temperature in the dark for 30 min, and absorbance was acquired at 517 nm. Scavenging activity was estimated through the Trolox Equivalent Antioxidant Capacity (TEAC) value [94].

##### FRAP Assay

The FRAP assay was conducted by incubating 10 μL of a sample solution (500 mg of dry sample in 10 mL of methanol) with 180 μL of FRAP solution that contains acetate buffer, TPTZ, and FeCl_3_. After 60 min of incubation at room temperature in darkness, the absorbance was acquired at 593 nm; ascorbic acid was used as the standard [95].

##### ORAC Assay

The ORAC assay was performed according to the AOAC 2012.23 Method (Official Methods of Analysis, 21st Edition, 2019 [96]). Previously, the sample was pretreated by dissolving 500 mg of dry material in 20 mL of solvent, using both hydrophilic (acetone–water) and lipophilic media (acetone). The test involved mixing 25 μL of the sample solution with 150 μL of fluorescein sodium salt, followed by incubation at 37 °C for 30 min in darkness. After this initial incubation, 25 μL of 2,2′-azobis(2-amidino-propane) dihydrochloride (AAPH) solution was added, and fluorescence was recorded for 60 min at an excitation wavelength of 485 ± 20 nm and an emission wavelength of 530 ± 25 nm. Standard solutions of Trolox were used to calculate the Oxygen Radical Absorbance Capacity (ORAC) values of the algae [81].

### 3.6. Statistical Data Analysis

The results of antioxidant tests and polyphenol quantification were statistically analyzed using Python software 3.10.10. Each antioxidant activity assay was performed three times from the same extract in order to determine their reproducibility. Statistical analyses were achieved with the Python software. First, normality and equal variances tests were carried out to assess if data had a normal distribution and if the variance of the groups was similar. Subsequently, a one-way ANOVA was used to test differences in chemical parameters and activity between species. When the model was significant (*p* < 0.05), Tukey’s HSD test was used as post hoc.

## 4. Conclusions

Macroalgae represent valuable natural resources for bioprospecting and sustainable development, owing to their high biomass yield and potential as raw materials for industries such as nutrition, medicine, agriculture, and cosmetics. Despite the numerous studies on macroalgae, those found in Colombia remain largely unexplored as a source of functional ingredients. This study contributes to unlocking the potential of these and other seaweeds for the development of aquaculture enterprises, thereby supporting local communities in benefiting from these resources by promoting bioeconomic diversification in Colombia and other tropical countries through the sustainable use of marine biodiversity.

As a result, functional components such as fatty acids, polysaccharides, and antioxidants were detected in the three macroalgae. More specifically, although all of them showed similar results, the content of mono and polyunsaturated fatty acids was potentially higher in *Sargassum* algae than in the algae of the genus *Acanthophora.* Furthermore, regarding the partial state of purification of the polysaccharides analyzed, the lack of monosaccharide composition, linkage, sulfate patterns, and a deeper structural analysis, the spectroscopic chemical analyses by NMR, FT-IR, and SEC were useful to identify structural features consistent with carrageenan, alginate, and fucoidan in the aqueous extracts of *A. spicifera*, *S. ramifolium,* and *S. fluitans*, respectively. These findings indicate that the algae may have diverse application potentials since these polymers have shown a diverse group of biological effects and uses. This difference has been observed in the total polyphenol composition of the algae and their antioxidant potentials. Among them, the alga *S. ramifolium* showed the most favorable antioxidant profile, followed by *S. fluitans,* and *A. spicifera*.

While the results suggest potential applications in several fields, including the biomedicine, cosmetics, functional foods, and nutraceutical industry, the effect of seasonal variation on the composition of polysaccharides, fatty acids, and antioxidant compounds needs to be thoroughly analyzed. This is essential to ensure the continuous valorization of different species and to support further research into the chemical composition of seaweeds, the standardization of methodologies for biomass transformation to add value, and the confirmation of the functionality of both known and novel compounds.

## Figures and Tables

**Table 1:** Fatty acid content in g/100 g DW of the studied Colombian macroalgae.

Fatty Acid	*Acanthophora spicifera*	*Sargassum* *ramifolium*	*Sargassum* *fluitans*
14:0	0.05	0.04	0.02
15:0	0.01	ND ^1^	ND ^1^
16:0	0.33	0.31	ND ^1^
16:1ω7	0.02	0.04	0.01
18:0	ND ^1^	0.01	ND ^1^
18:1ω9	0.10	0.13	0.04
18:2ω6	0.02	0.03	0.02
18:3ω3	ND ^1^	0.01	ND ^1^
20:4ω6	ND ^1^	0.03	0.01
22:0	ND ^1^	0.01	ND ^1^

^1^ ND: not detected or <0.01 g/100 g DW.

**Table 2:** Molecular weight of the polysaccharides in the aqueous extracts.

Sample	Rt	Log MW	Mp (kDa) *
*A. spicifera*	16.30–18.60	5.204	92–160
*S. ramifolium*	15.90	5.246	176
*S. fluitans*	15.70	5.267	185

* y = −0.105x + 6.9155 (r^2^ = 0.9934).

**Table 3:** Total polyphenol compounds and antioxidant activity via DPPH, ORAC, FRAP, and ABTS tests.

Alga	TPC	DPPH	ORAC	FRAP	ABTS
*A. Spicifera*	366.04 ± 6.76	39.17 ± 1.73	541.09 ± 21.89	49.19 ± 0.98	283.42 ± 5.67
*S. ramifolium*	936.67 ± 18.25	102.84 ± 3.34	3362.67 ± 99.85	113.24 ± 6.18	1830.69 ± 88.62
*S. fluitans*	449.43 ± 6.06	57.69 ± 2.42	1898.04 ± 65.70	128.74 ± 2.55	1237.45 ± 18.08

TPC values are expressed in milligrams of Gallic Acid Equivalent per 100 g of dried sample (mg GAE/100 g dw); FRAP in milligrams of Ascorbic Acid Equivalent per 100 g of dried sample (AAE/100 g dw); and ABTS, ORAC, and DPPH in milligrams of Trolox Equivalent per 100 g of dried sample (TE/100 g dw). Each value is presented as mean value (*n* = 3) ± standard deviation.

**Table 4:** One-way ANOVA for TPC and antioxidant activity.

Statistical Measure	Test				
	TPC	FRAP	ABTS	DPPH	ORAC
F-value	482.57	351.23	668.87	482.57	1214.40
*p*-Value *	2.36 × 10^−7^	6.07 × 10^−7^	8.90 × 10^−8^	2.36 × 10^−7^	1.50 × 10^−8^

* *p* < 0.05.

**Table 5:** Multiple Comparison of Means—Tukey HSD.

Groups	Statistical Measure *	Test				
		TPC	FRAP	ABTS	DPPH	ORAC
*A. spicifera* –*S. fluitans*	Mean difference	83.37	79.56	954.08	18.52	1357.4
	*p*-Value	0.0003	0.0	0.0	0.0003	0.0
*A. spicifera* –*S. ramifolium*	Mean difference	570.71	64.15	1548.64	63.68	2822.26
	*p*-Value	0.0	0.0	0.0	0.0	0.0
*A. spicifera* –*S. fluitans*	Mean difference	487.34	−15.41	594.56	45.16	1464.86
	*p*-Value	0.0	0.0069	0.0	0.0	0.0

* α < 0.05.

**Table 6:** Codification of the studied macroalgae.

Species	Group	Order	Family	Collection Date	Collection Site	Voucher Number
*A. spicifera*	Red macroalgae	Ceramiale	Rhodomelacea	28 February 2021	Santa Marta	JIW00005017
*S. ramifolium*	Brown macroalgae	Fucales	Sargassaceae	1 March 2021	Guajira	JIW00004940
*S. fluitans*		Fucales	Sargassaceae	1 February 2021	San Andrés	JIW00004945

## Data Availability

The original data presented in the study is included in the article; further information can be requested directly from the corresponding author.

## Supplementary Materials

The following supporting information can be downloaded at: https://www.mdpi.com/article/10.3390/molecules30163333/s1, Table S1: Calculation formulas used for fatty acids detected; Table S2: Conversion factors for conversion of FAMEs to their corresponding fatty acids detected in macroalgae; Table S3: Experimental data of fatty acid methyl esters from macroalgae samples.

## Abbreviations

The following abbreviations are used in this manuscript:

TPC	Total phenolic compounds
ORAC	Oxygen Radical Absorbance Capacity
FRAP	Ferric-reducing antioxidant power
TEAC	Trolox equivalent antioxidant capacity
ABTS	2,2-azino-bis-3-ethylbenzothiazoline-6-sulphonic acid
GAE	Gallic acid equivalent
NMR	Nuclear magnetic resonance
HPLC	High performance liquid chromatography
FT-IR	Fourier-transform infrared spectroscopy
FAME	Fatty acid methyl ester
