# Supplementary material for: Chemical Exploration of Polysaccharides, Fatty Acids, and Antioxidants as Functional Ingredients from Colombian Macroalgae Acanthophora spicifera, Sargassum ramifolium, and Sargassum fluitans
Source: Molecules. 2025 Aug 10;30(16):3333. doi: 10.3390/molecules30163333 (PMC12388559; doi:10.3390/molecules30163333)
Supplement: Supplementary file 1 [file molecules-30-03333-s001.zip › molecules-3736885-supplementary.pdf]

Supplementary materials

Chemical Exploration of Polysaccharides, Fatty Acids, and Antioxidants as Functional Ingredients from Colombian Macroalgae *Acanthophora spicifera*, *Sargassum ramifolium*, and *Sargassum fluitans*

Table S1. Calculation formulas used for fatty acids detected

Formulas
$R_i = \frac{\mathrm{Ps}_i}{\mathrm{Ps}_{C11:0}} \times \frac{W_{C11:0}}{W_i}$
$W_{FAMEi} = \frac{Pt_i \times Wt_{C11:0} \times 1.0067}{Pt_{C11:0} \times R_i}$
$W_i = W_{FAMEi} \times f_{FAi} \times 100)/W_{testportion}$

R_i= response factor

Ps_i = peak area of individual fatty acid in mixed FAMEs standard solution;

$Ps_{C11:0}$ = peak area of $C_{11:0}$ fatty acid in mixed FAMEs standard solution;

$W_{C11:0}$ = weight of internal standard in mixed FAMEs standard solution;

W_t = weight of individual FAME in mixed FAMEs standard solution.

W_{FAMEi} = amount of individual fatty acids in test portion

Pt_i = peak area of fatty acid *i* in test portion

$Wt_{C11:0}$ = weight of $C_{11:0}$ internal standard added to test portion in g

1.0067 = conversion of internal standard from triglyceride to FAME

$Pt_{C11:0}$ = peak area of $C_{11:0}$ internal standard in test portion

W_i = weight of each fatty acid

f_{Ai} = conversion factor for FAMEs to corresponding fatty acids (*see* Table S2.).

$W_{test\ portion}$ = weight of test portion in g.

Table S2. Conversion factors for conversion of FAMEs to their corresponding fatty acids detected in macroalgae

FAME	f_{Ai} [a]
**C11:0**	0.9300
**C14:0**	0.9421
**C15:0**	0.9453
**C16:0**	0.9481
**C16:1**	0.9477
**C18:0**	0.9530
**C18:1**	0.9527
**C18:2**	0.9524
**C18:3**	0.9520
**C20:4**	0.9560
**C22:0**	0.9604

[a] f_{Ai} is the conversion factor for FAMEs to corresponding fatty acids

Table S3. Experimental data of fatty acid methyl esters from macroalgae samples

Symbol	Name	RT (min)	Area			
			FAME-mix	*A. spicifera*	*S. ramifolium*	*S. fluitans*
C11:0	Methyl undecanoate	5.537	1064612	7305123	12485573	7754055
C14:0	Methyl myristate	8.678	3569807	373828	460259	168593
C15:0	Methyl pentadecanoate	10.026	1807737	46863	43629	13736
C16:0	Methyl palmitate	11.561	4910203	3707333	4897377	1741046
C16:1	Methyl heptadecanoate	12.185	576862	35486	154362	24669
C18:0	Methyl stearate	15.015	3203378	1218958	1412384	847275
C18:1	(cis-9) Methyl oleate	15.524	1070287	318226	548723	199752
C18:2	(all-cis-9,12) Methyl linoleate	16.654	731586	56735	132741	63160
C18:3	(all-cis-9,12,15) Gamma-Methyl linolenate	18.132	899720	13886	61837	8608
C20:4	(all-cis-5,8,11,14) Gamma-Methyl linolenate	21.691	695163	11106	128334	26135
C22:0	Methyl behenate	22.704	3014802	11125	58886	27402
